# Accidental Intrathecal Morphine Overdose During the Surgery: A Case Report and Literature Review

**DOI:** 10.7759/cureus.77377

**Published:** 2025-01-13

**Authors:** Dafni Andromachi Antonaki, Emmanouil Stamatakis, Giolanda Varvarousi, Dimitrios Valsamidis

**Affiliations:** 1 Department of Anesthesiology, Laiko General Hospital of Athens, Athens, GRC; 2 Department of Anesthesiology, Alexandra General Hospital of Athens, Athens, GRC

**Keywords:** accidental administration, accident investigation, chronic pain management, intrathecal morphine therapy, intrathecal opioids, morphine overdose, postoperative analgesia

## Abstract

Neuraxial anesthetic techniques have become part of the multimodal analgesia approach for gynecologic surgeries. Specifically, intrathecal morphine is one of the opioids most commonly used for prolonged pain management postoperatively. However, doses above a certain threshold pose a greater risk of adverse effects, especially respiratory complications. This case highlights the challenges faced during the management of an accidental administration of a tenfold dose of morphine in the subarachnoid space for a patient subjected to major gynecologic surgery. Surprisingly, yet to our advantage, the patient exhibited no evidence of the anticipated adverse effects and no further intervention was required. A key indicator that was crucial in identifying the mistake was a diminution in the minimal alveolar concentration (MAC) of the anesthetic agent used.

## Introduction

Intrathecal administration of morphine was initially introduced in 1979 by Dr. Behar and his associates. This pioneering method allowed for significant pain relief using smaller doses compared to systemic administration, thereby marking a milestone in the field of pain control [1,2]. Since then this technique has been widely used by anesthesiologists also for many types of surgical procedures such as cesarean sections and orthopedic, spinal, abdominal, and urological surgeries.

Intrathecal morphine has favorable effects on pain management for a longer period of time than other intrathecal opioids and overall reduces the additional use of systemic opioids [1]. The standard doses for intrathecal morphine can vary depending on the type of surgery and the individual patient's needs, but generally, the standard dosing usually ranges from 0.1 to 0.2 mg (100 to 200 mcg). The maximum recommended dose for intrathecal morphine generally does not exceed 0.3 mg (300 mcg) in most clinical settings. Doses above this threshold increase the risk of adverse effects such as respiratory depression, pruritus, nausea, and vomiting. Finding the right balance between effective analgesia and minimal respiratory depression is the ultimate objective and this case report aims to make even a minor contribution towards this goal [3-5].

We present a case of an accidental intrathecal morphine overdose in a patient undergoing exploratory laparotomy for an ovarian mass, along with a review of the relevant literature.

## Case presentation

A 55-year-old female patient with a history of sinus tachycardia, currently managed with atenolol, was scheduled for a routine exploratory laparotomy to investigate and resect an ovarian mass identified during standard abdominal imaging. The patient had no other medical history. The anesthesia team initially planned to administer 100 mcg of morphine combined with a local anesthetic intrathecally for both operative and postoperative pain management, using a combined epidural-spinal technique. Following this, the patient was scheduled to receive general anesthesia, according to the hospital protocol for both general and neuraxial anesthesia. However, during the preoperative visit, the patient, due to a previous adverse experience with epidural anesthesia during labor, declined the epidural. After informed consent, the team proceeded with spinal anesthesia in combination with general anesthesia as planned.

In the operating room, two 18-gauge IV lines were placed on the upper limbs and the patient was placed in the left lateral decubitus position for the neuraxial block procedure. The L3-L4 interspace was identified using Tuffier's line and by palpating the spinous processes. A pencil point, 25-gauge, Whitacre needle (BD, Becton, Dickinson and Company, Franklin Lakes, USA) was used, and the subarachnoid space was accessed utilizing the paramedian approach. A 1 mL ampoule of 1% morphine was opened and aseptically diluted in a 10 mL syringe with 9 mL of 0.9% sodium chloride, resulting in a 0.1% morphine solution (1000 mcg/mL). It was intended to further dilute this preparation in a second sterile syringe to achieve a 0.01% morphine concentration (100 mcg/mL). Nevertheless, the process of the second dilution was not completed due to an error in communication within the anesthesia team. Consequently, 1.5 mL of the initial solution, containing 1.5 mg (1500 mcg) of morphine, was inadvertently administered into the subarachnoid space in addition to 2 mL of 0.5% hyperbaric bupivacaine.

The subsequent procedure followed standard protocols. Initially, a pre-induction bolus of antiemetics was administered, consisting of metoclopramide (10 mg), cimetidine (200 mg), ondansetron (4 mg), and dexamethasone (8 mg). Anesthesia was then induced using fentanyl (100 mcg), propofol (140 mg), and rocuronium (50 mg). The patient was successfully intubated without complications. Maintenance of anesthesia was achieved with desflurane, initially targeting a minimum alveolar concentration (MAC) of approximately 1. Standard physiological monitoring included heart rate, non-invasive blood pressure (BP), oxygen saturation, and body temperature. Additionally, urine output was monitored via a Foley catheter. Depth of anesthesia was assessed using a bispectral index (BIS) monitor, and respiratory parameters were continuously monitored, including capnography, fraction of inspired oxygen (FiO2), minute ventilation (MV), peak inspiratory pressure (PIP), lung compliance, and positive end-expiratory pressure (PEEP). Neuromuscular blockade was assessed using train-of-four (TOF) monitoring. The patient received 2 gm of cefoxitin and 500 mg of metronidazole for surgical site infection (SSI) prophylaxis before the surgical incision and in the course of multimodal analgesia, 1 gram of paracetamol and 50 mg of dexketoprofen were administered. Additionally, the fluid balance was maintained using crystalloid solutions, specifically, Ringer’s lactate at a dosage of 15 mL/kg, and a 20% human albumin solution was administered gradually to support intravascular volume in view of the drainage of more than three liters of ascitic fluid from the peritoneal cavity.

During the surgical procedure, the patient remained hemodynamically stable, with BP measurements ranging from systolic/diastolic blood pressure (SAP/DAP) of 95/55 mmHg to 110/65 mmHg, showing minimal fluctuations. The heart rate was maintained between 60 to 75 beats per minute. Throughout the surgery, the BIS indicated deep sedation with values of 43, 45, 40, and 50. Interestingly, the MAC of the inhaled anesthetic agent that was titrated according to BIS was lower than expected, measuring at 0.6, which suggests a deeper anesthetic effect than anticipated by the dosage. Upon making this observation, the anesthesiologist inspected the diluted morphine syringes and identified the error. Immediately after the discovery, the error was communicated to the rest of the operating room team, including the nurse anesthetist and the surgeon. This timely notification was crucial for ensuring that all team members were prepared to manage any potential adverse events arising from the incident.

The surgical procedure lasted three hours, and upon its conclusion, the anesthetic team opted to awaken the patient. Consequently, the patient was successfully extubated following the reversal of neuromuscular blockade with sugammadex when the TOF value was 0.9 and the anesthetic MAC value was 0.1. Upon awakening, the patient was fully oriented, exhibited proficient communication skills, achieved a Glasgow Coma Scale (GCS) score of 15, and maintained a respiratory rate (RR) of more than 16 breaths per minute. Following the post-anesthesia care unit (PACU), the patient was transferred to the high-dependency unit (HDU) with explicit instructions for constant monitoring of vital signs, particularly RR, oxygen saturation (SpO2), and BP. The care team was also directed to assess the GCS every two hours and to monitor for the emergence of nausea, vomiting, or pruritus. An emergency airway trolley was available and ready for use and instructions for naloxone use on the indications of respiratory distress.

Fortunately, the patient did not experience any of the anticipated adverse effects. After a 24-hour observation period, she was transferred to the gynecological oncology unit to continue her hospitalization and rehabilitation.

## Discussion

Intrathecal opioid use has been popularized for abdominal gynecological surgeries due to the rising numbers of the laparoscopic technique, the relatively simpler administration, low cost, and strong analgesic effect [6]. Morphine is the most commonly used opioid for subarachnoid injection with its most prominent pharmacological properties being the long-lasting analgesic effect and deep pain relief. Intrathecal morphine use for perioperative pain control has become a standard practice in many institutes including our own. It has been shown to significantly shorten the length of hospital stay and the need for postoperative intravenous opioids [7]. It has been proposed that the ideal dosage varies based on the surgical context and that there is a ceiling analgesic effect above which the risk of adverse effects outweighs the benefits of enhanced analgesia [8]. The most critical adverse effect after intrathecal morphine use is respiratory depression, which clinically occurs as a diminished RR and depth. Even though bradypnea is a potentially severe condition on its own, the delayed onset is also a major concern as it can occur up to 48 hours after the administration of morphine. This hazard poses the need for continuous respiratory and basic monitoring of the patient who receives spinal morphine for at least 24 hours in a HDU [3,9-11].

Other adverse effects that have been described in the literature are pruritus, nausea, and vomiting, urinary retention, somnolence, hypothermia, and rigor. Although these symptoms can be bothersome for patients and negatively impact the overall postoperative experience, they are typically benign and do not pose a threat to overall health or life [10,11].

Throughout medical literature, there have been numerous reports documenting cases of accidental spinal administration of morphine. A brief review of reported cases involving intrathecal morphine overdose highlights several critical incidents and management strategies. These cases predominantly involve errors in drug administration, either through pump refill mistakes, syringe mix-ups, or incorrect drug vial usage, resulting in doses significantly exceeding the therapeutic range [12-25]. The largest reported overdose involved a 510 mg (in 35 mL) bolus of morphine, far surpassing the typical doses used in clinical settings. This case was further complicated by status epilepticus in addition to the anticipated adverse effects [20]. As reported in certain other cases, these accidents can be perplexed by total spinal anesthesia where airway management is inherently required [16,24]. Management of these cases primarily involved the administration of naloxone, both as continuous infusions and bolus doses, to reverse the opioid effects. This intervention was described in almost all of the cases, leading to good patient outcomes, as it reversed the adverse effects of the opioid overdose but not the analgesic effect. Additional strategies included the aspiration and irrigation of cerebrospinal fluid (CSF) to remove the excess drug and dilute the remaining morphine in the CSF [21-23]. Mechanical ventilation for respiratory support was needed when respiratory depression was not reversible with naloxone. BP management was crucial in patients presenting with severe hypertension or hypotension (Tables 1, 2) [13,21,22]. The highest doses administered were observed during intrathecal pump refills for chronic pain management. In these cases, the patients differed from the surgical patients, who were opioid naive. In these cases of giga-dose errors, a distinguishing factor compared to other patients (the surgical group) was the occurrence of seizures, likely attributable to morphine lowering the seizure threshold [26].

**Table 1:** Accidents Occurring in the Operating Room/Morphine for Postoperative Analgesia I/MV: Intubation/mechanical ventilation; ICU: Intensive care unit; CSF: Cerebrospinal fluid; RD: Respiratory depression; AF: Atrial fibrillation; UR: Urinary retention; S: Somnolence; PACU: Postanesthesia care unit; LSCR: Laparoscopic segmental colonic resection; NS: Normal saline

Author(s), Year	Age/Sex	Indication	Dose (mg)	Error Type	Symptoms	Naloxone Use	Additional Treatment
Perrot G et al., 1983 [19]	42/M	Femoral derotation osteotomy	8mg	Syringe mix-up	RD/vomiting/UR	1.2mg over 5 hours, then 9.6mg over 24hrs	ICU/bladder catheterisation
Pomonis SP et al., 1986 [15]	90/F	Femoral fracture	15mg	Error in dilution	S/ RD	Nalorphine	I/MV
Pomonis SP et al., 1986 [15]	82/F	Femoral fracture	15mg	Error in dilution	S/RD	Nalorphine	I/MV
Pomonis SP et al., 1986 [15]	61/M	Patella fracture	15mg	Error in dilution	S/RD	Nalorphine	I/MV
Pomonis SP et al., 1986 [15]	78/M	Femoral fracture	15mg	Error in dilution	S/RD	Nalorphine	I/MV
Kaiser KG and Bainton CR, 1987 [23]	81/M	Left hemicolectomy	5mg	Syringe mix-up	S	No	CSF drainage and replacement with NS
Kim SC et al., 1990 [16]	39/F	Total Abdominal Hysterectomy	3mg	Intended epidural administration	Total spinal anesthesia/RD	0.4mg bolus/0.4 mg/100 mL/hr infusion/0.2mg bolus	I/MV
Cannesson M et al., 2002 [18]	31/F	Cesarean section	25mg	Vial mix-ups	S/pruritus/nausea	0.4 mg bolus,then 80 mcg/hr, then 200 mcg/hr	ICU, metoclopramide
Gerancher JC and Nagle PC, 2008 [17]	45/F	Exploratory laparotomy	7.5mg	Vial mix-ups	S/nausea	40 mcg/hr, then up to 140 mcg/hr	PACU
de Morais BS et al., 2008 [14]	45/M	Reconstruction of the anterior cruciate ligament of the left knee	4mg	Vial mix-ups	Vomiting/diaphoresis/AF/UR	0.4mg bolus, 0.1 to 0.2mcg/kg/hr infusion	PACU, bladder catheterisation/ondansetron/amiodarone
Kanazawa S and Okutani R, 2015 [12]	32/F	Cesarean section	1mg	Error in dilution	Hypothermia/nausea/RD	0.2 mg over 10 minutes	Monitoring
Koning MV et al., 2002 [13]	80/F	LSCR	5mg	Pharmacy compounding error	S/RD	100 mcg bolus, then at 10 hours and at 22 hours	Monitoring
Koning MV et al., 2002 [13]	73/M	LSCR	5mg	Pharmacy compounding error	None	No	None
Koning MV et al., 2002 [13]	72/M	LSCR	4mg	Pharmacy compounding error	Hypotension	No	ICU/norepinephrine infusion
Koning MV et al., 2002 [13]	74/F	LSCR	5mg	Pharmacy compounding error	S/RD	Infusion for 36 hours	ICU/supplemental oxygen
Koning MV et al., 2002 [13]	68/M	LSCR	3mg	Pharmacy compounding error	None	No	None
Koning MV et al., 2002 [13]	63/M	LSCR	3mg	Pharmacy compounding error	S/RD	100mcg	Monitoring
Samara E et al., 2023 [25]	64/M	Sigmoidectomy	1mg	Wrong dilution	None	Continuous drip 0.5 mcg/kg/hr	PACU

**Table 2:** Accidents Occurring During Pump Refills/Morphine for Chronic Pain Management SE: Status epilepticus; I/MV: Intubation/mechanical ventilation; ICU: Intensive care unit; CSF: Cerebrospinal fluid; RD: Respiratory depression

Author(s), Year	Age/Sex	Morphine Dose (mg)	Error Type	Symptoms	Naloxone Use	Additional Treatment
Sauter K et al., 1994 [22]	45/F	450	Pump refill error	Agitation, hypertension, seizure, SE	8 mg bolus, then 20 mg/hr	ICU, I/MV, CSF drainage, nitroprusside
Groudine SB et al., 1995 [21]	56/F	250	Syringe mix-up	Hypotension, agitation, RD, seizure	100 mcg/hr for 5 hours	ICU, ephedrine, I/MV, CSF drainage and irrigation
Yilmaz A et al., 2003 [20]	47/F	510	Pump refill error	Agitation, double vision, headache, seizure	0.4 mg/hr	Diazepam, I/MV, thiopental
Sidlak AM et al., 2019 [24]	74/F	100	Intrathecal pump error	Hypotension/somnolence/SE	0.8 mg bolus	Hospital transfer, I/MV

These instances often serve as critical learning points for the medical community, emphasizing the need for meticulous procedural protocols and safeguards to prevent such errors. In comparison to other reported cases, this patient exhibited no signs of morphine overdose, indicating the broad range of morphine dosages which may or may not result in adverse effects. In addition to that, we opted to closely monitor the patient rather than administer naloxone preemptively. Ultimately, there was no need for naloxone administration. However, as demonstrated in several studies, a reduction in the MAC of the volatile anesthetic during surgery was observed, which ultimately raised suspicion and led the anesthesiologist to identify the error [27-29].

Reports of accidental administration of drugs typically discuss the circumstances leading to the error, the immediate management of the overdose, and the outcomes for the patients involved. A systematic review of quantitative and qualitative evidence conducted by Keers et al. states the most prevailing causes for error- or violation-provoking conditions are inadequate written communication (prescriptions, documentation, transcription), problems with medicine supply and storage (pharmacy dispensing errors and ward stock management), high perceived workload, problems with ward-based equipment (access, functionality), patient factors (availability, acuity), staff health status (fatigue, stress) and interruptions/distractions during drug administration [30].

In summary, intrathecal morphine, while highly effective for pain management, carries the risk of significant side effects and potential for dosing errors, as highlighted by various case reports. Early detection of such errors through cautious monitoring, including parameters like MAC reduction, can prevent serious complications. Additionally, ensuring solid communication protocols among all the members of the anesthetic team is essential for minimizing the risk of accidental overdoses and optimizing patient safety and overall recovery.

## Conclusions

This case report highlights the critical need for alert intraoperative monitoring, where an unexpectedly low MAC of the volatile anesthetic served as a primary indicator of a severe intrathecal morphine overdose. Regardless of the substantial dose, the absence of severe consequences in this patient indicates the range in opioid pharmacodynamics and argues for the necessity of further research to potentially refine dosing guidelines. Furthermore, this case emphasizes the importance of closed-loop communication within the anesthetic team to avert such errors, reassuring that all steps in medication preparation and administration are ensured and confirmed. The verdicts from this case constitute not only a reminder of the likely effects of medication errors but also a confirmation of the persistence of proper monitoring protocols in safeguarding patient consequences.
